# Reporting and analysing ethnicity in populational health data and linkage research: A bibliographical review

**DOI:** 10.23889/ijpds.v8i2.2229

**Published:** 2023-09-14

**Authors:** Joseph Lam, Robert Aldridge, Ruth Blackburn, Katie Harron

**Affiliations:** 1 UCL Great Ormond Street Institute of Child Health, London, United Kingdom; 2 UCL Institute of Health Informatics, London, United Kingdom

A good understanding of age, when individuals develop individual conditions and subsequently become multimorbid, would provide evidence to support better prevention, detection, and management of multiple long-term conditions. The study aimed to assess the age at onset, incidence, and prevalence of 31 long-term conditions and multimorbidity in Fife and Tayside, Scotland by sex and socioeconomic status.

We linked routinely collected health records of 431,772 patients aged 25 years or higher on January 1st, 2000 till December 31st, 2018 from several database. Age of first report of the 31 Elixhauser long-term conditions was assessed.

Median age at the onset of conditions within the population ranged from 39 years for AIDS/HIV to 73 years for fluid and electrolyte disorders and 75 years for renal failure. This was mostly delayed among the least deprived than most deprived people, up to 14 years for psychoses and AIDS/HIV. People with the highest deprivation in both sexes had earlier onset of all conditions except for blood loss anaemia where most deprived males had three years delay. Median age at the onset of multimorbidity and complex multimorbidity was 67 years (68 for females vs 66 for males; 68 for most deprived vs 73 for least deprived) and 71 years (73 for females vs 70 for males; 73 for most deprived vs 78 for least deprived) respectively. The yearly incidence of both multimorbidity and complex multimorbidity rose steadily from 1.6% and 0.2% in 2000, peaked in 2015 (3.2% vs 1.4%), declined to 2.9% and 1.3% respectively in 2018 while the prevalence grew from 1.6% and 0.2% to 27.2% and 10.1% over the same period.

A number of conditions had earlier onset while others were delayed. The onset of conditions is generally about five years earlier among most deprived people than the least deprived just as multimorbidity and complex multimorbidity is higher and earlier among the most deprived. This information could help drive health system policy, management, intervention, and allocation of resources.

